# Changes in birth outcomes and utilization of prenatal care during the COVID-19 pandemic in 2020: a secondary analysis of vital statistics in Colombia

**DOI:** 10.1186/s12887-023-04027-9

**Published:** 2023-05-12

**Authors:** Carol C. Guarnizo-Herreño, Giancarlo Buitrago, George L Wehby

**Affiliations:** 1 Facultad de Odontología, Universidad Nacional de Colombia, Bogotá, Colombia; 2 Facultad de Medicina, Universidad Nacional de Colombia, Bogotá, Colombia; 3 Hospital Universitario Nacional de Colombia, Bogotá, Colombia; 4 Department of Health Management and Policy, College of Public Health, University of Iowa, Iowa, USA; 5 National Bureau of Economic Research, Cambridge, MA USA

**Keywords:** Prenatal care, Birth weight, Perinatal death, COVID-19, Colombia

## Abstract

**Background:**

Birth outcomes could have been affected by the COVID-19 pandemic through changes in access to prenatal services and other pathways. The aim of this study was to examine the effects of the COVID-19 pandemic on fetal death, birth weight, gestational age, number of prenatal visits, and caesarean delivery in 2020 in Colombia.

**Methods:**

We conducted a secondary analysis of data on 3,140,010 pregnancies and 2,993,534 live births from population-based birth certificate and fetal death certificate records in Colombia between 2016 and 2020. Outcomes were compared separately for each month during 2020 with the same month in 2019 and pre-pandemic trends were examined in regression models controlling for maternal age, educational level, marital status, type of health insurance, place of residence (urban/rural), municipality of birth, and the number of pregnancies the mother has had before last pregnancy.

**Results:**

We found some evidence for a decline in miscarriage risk in some months after the pandemic start, while there was an apparent lagging increase in stillbirth risk, although not statistically significant after correction for multiple comparisons. Birth weight increased during the onset of the pandemic, a change that does not appear to be driven by pre-pandemic trends. Specifically, mean birth weight was higher in 2020 than 2019 for births in April through December by about 12 to 21 g (p < 0.01). There was also a lower risk of gestational age at/below 37 weeks in 2020 for two months following the pandemic (April, June), but a higher risk in October. Finally, there was a decline in prenatal visits in 2020 especially in June-October, but no evidence of a change in C-section delivery.

**Conclusions:**

The study findings suggest mixed early effects of the pandemic on perinatal outcomes and prenatal care utilization in Colombia. While there was a significant decline in prenatal visits, other factors may have had counter effects on perinatal health including an increase in birth weight on average.

**Supplementary Information:**

The online version contains supplementary material available at 10.1186/s12887-023-04027-9.

## What is known

Perinatal outcomes could be affected by the COVID-19 pandemic through changes in access to prenatal services and other pathways.

Evidence to date on this issue paints a mixed picture, with some studies reporting worse outcomes while others finding no changes or better outcomes. Little is known about the pandemic effects on perinatal outcomes in South American populations.

**Supplementary Information:**

The online version contains supplementary material available at 10.1186/s12887-023-04027-9.

## What is new

- Our study suggests an increase in mean birth weight and decline in miscarriage risk but also fewer prenatal visits in 2020 following the pandemic start in Colombia.
- There was a lower risk of gestational age at/below 37 weeks for two months in 2020 but overall little evidence for an effect on C-sections.

**Supplementary Information:**

The online version contains supplementary material available at 10.1186/s12887-023-04027-9.

## Introduction

The COVID-19 pandemic has affected population health worldwide in multifaceted and complex ways. Besides the direct consequences of SARS-Cov-2 infections, the social, economic, mobility, and health care changes from the pandemic and the policies enacted to mitigate it have affected health outcomes across countries and societies [1–3]. These changes are also closely related to perinatal health. Although evidence is emerging on the pandemic effects on perinatal health outcomes in some countries,[4, 5] little is known about these effects in South American populations. We address this gap by providing evidence on changes in perinatal health outcomes and prenatal care utilization in 2020 in Colombia.

The lockdowns, school closures, unemployment rise, and restrictions on accessing (or personal reluctance to use) health services may all have had adverse effects on perinatal health by increasing maternal stress and reducing prenatal care use [4, 6, 7]. Similar to many countries, Colombia implemented strict lockdowns and travel restrictions starting in March 2020 enforced by both national and local governments. Access and provision of health care was largely restricted focusing on emergency services and delaying preventive, routine, and less urgent services. Unlike high-income countries where income support was provided by governments to counter income loss from unemployment, such programs were largely unavailable or very limited in Colombia, particularly early during the pandemic. On the other hand, the pandemic and the policies enacted to mitigate it might have had some beneficial effects on perinatal health from reduced maternal exposure to air pollution, occupational health hazards, and job stress [6, 8].

The evidence thus far on the pandemic effects on maternal, fetal and neonatal outcomes paints a mixed picture, with some studies reporting worse outcomes while others finding no changes or better outcomes [4, 9–15]. A systematic review of studies published through January 2021 found global increase in maternal deaths, stillbirths, ruptured ectopic pregnancies, and maternal depression [4]. No significant changes were identified in preterm births, low birthweight, gestational diabetes, and 5-minute Apgar score below 7 [4]. Multiple recent studies, mainly from high-income countries, have also reported no changes in preterm births, stillbirths, perinatal/neonatal mortality, and caesarean delivery [10–12, 14, 15] while some reported a decline in preterm births, low birthweight, and stillbirths [9–11, 13, 14]. Furthermore, the above mentioned systematic review found no significant changes in labour induction or delivery mode;[4] and a study in the US showed no changes in caesarean section rates [11]. In contrast, there is evidence, mostly from low- or middle-income countries, on declines in prenatal care access and utilization including both delayed initiation and fewer visits [16–18].

The pandemic effects on perinatal outcomes could vary between countries due to differences in economic consequences and government income support interventions, mitigation policies, and social, economic, and health care infrastructures [10, 19, 20]. Moreover, although there is a growing literature on this question, most studies are from high-income countries and less is known about these effects in low- and middle-income settings.

This study is among the first to examine the pandemic effects on a range of perinatal health outcomes and utilization of prenatal care in a South American country. Using data on virtually the universe of births in Colombia between 2016 and 2020, we examined month-by-month differences between 2019 and 2020 in multiple perinatal outcomes and utilization of prenatal care, and whether differences reflect pre-pandemic trends (2016–2019) or new changes during the pandemic in 2020. We used population-based data from birth certificate and death certificate records to study fetal death, birth weight, gestational age, number of prenatal visits, and caesarean delivery. Perinatal outcomes have shown associations with various health, developmental, and wellbeing outcomes during childhood and throughout the life course [21–23]. Number of prenatal visits was also included given its importance for perinatal health and since access to care was affected by the pandemic [4, 24–26].

## Materials and methods

To estimate the effects of the COVID-19 pandemic on perinatal outcomes and prenatal care utilization, we compared those outcomes before and during the pandemic period. We include data from 2016 to 2019 to evaluate trends in outcomes before the pandemic. In Colombia, lockdowns and other measures to mitigate the spread of COVID-19 were in place beginning mid-March 2020. We compared outcomes separately for each month of the year during 2020 with the same months in 2019. Estimates from 2019 were also compared with the preceding 3 years to assess pre-pandemic trends. We chose this month-specific comparison approach since births in 2020 were exposed to different durations of the pandemic depending on birth month and given potential seasonal variation in the study outcomes.

### Data sources and study population

We used two population-based registers available in Colombia, the birth and fetal death certificate records datasets. The coverage of birth certificates for Colombia in 2015 was 97% (based on the 2015 Colombian Demographic and Health Survey). On the other hand, the information on coverage of the death certificates is very outdated and no recent figure is available [21–23]. Birth and fetal death certificates are usually completed in health-care facilities where the birth/death occurred and are filled out by the health care provider who delivered the infant or pronounced the death. In areas with health care provider shortages, community health workers and nursing assistants can also fill out the certificates. The Colombian National Department of Statistics (DANE) oversees the data and makes it publicly available. For this analysis, we used the open-access databases of birth and death certificate records available at the DANE website (https://www.dane.gov.co). The birth and death certificates currently and previously used in Colombia are also available at the DANE website (https://www.dane.gov.co/index.php/estadisticas-por-tema/salud/nacimientos-y-defunciones).

All singleton pregnancies (n = 3,335,701) and singleton live births (n = 3,168,938) registered in Colombia during the 2016–2020 period were identified. The analytical sample included observations with complete data on the study variables (outcomes and covariates). The highest missing data rate was for maternal education with 4.3% missing data across the study period (Appendix Table 1). Due to missing data, we excluded from the sample 5.9% of pregnancies and 5.5% of live births. The missing data rate was fairly comparable across the study years, and very similar between 2019 and 2020, suggesting no differential missing data patterns by year that would bias the estimates of outcome changes during the pandemic (Appendix Tables 1 and 2). The final number of pregnancies and live births included in the analysis were 3,140,010 and 2,993,534 respectively.

**Table 1:** Risk of miscarriages (fetal deaths before 22 gestational weeks) across years with 2019 as the reference year

	January	February	March	April	May	June	July	August	September	October	November	December
Year	Regression estimates (Standard errors)											
2016	0.007**^¥^	0.007**^¥^	0.005**^¥^	0.004**^¥^	0.002	0.002	0.003**	0.004**^¥^	0.002	0.005**^¥^	0.003**	0.002*
	(0.001)	(0.001)	(0.001)	(0.001)	(0.001)	(0.001)	(0.001)	(0.001)	(0.001)	(0.001)	(0.001)	(0.001)
2017	0.008**^¥^	0.004**	0.005**^¥^	0.003*	0.001	0.001	0.001	0.003**	0.002	0.002*	0.002*	0.002
	(0.001)	(0.001)	(0.001)	(0.001)	(0.001)	(0.001)	(0.001)	(0.001)	(0.001)	(0.001)	(0.001)	(0.001)
2018	0.004**	0.005**^¥^	0.005**^¥^	0.005**^¥^	0.001	0.001	0.001	0.003**	0.002	0.002*	0.001	-0.001
	(0.001)	(0.001)	(0.001)	(0.001)	(0.001)	(0.001)	(0.001)	(0.001)	(0.001)	(0.001)	(0.001)	(0.001)
2019	Ref.	Ref.	Ref.	Ref.	Ref.	Ref.	Ref.	Ref.	Ref.	Ref.	Ref.	Ref.
2020	-0.000	0.000	-0.001	-0.004**	-0.004**^¥^	-0.003*	-0.009**^¥^	-0.004**^¥^	-0.003**^¥^	-0.001	-0.005**^¥^	0.001
	(0.001)	(0.001)	(0.001)	(0.001)	(0.001)	(0.001)	(0.001)	(0.001)	(0.001)	(0.001)	(0.001)	(0.001)
Observations	262,061	238,129	261,721	252,550	260,753	251,168	263,043	270,018	282,787	273,126	262,507	262,147

Estimates are rounded to the third decimal and are estimated relative to the total number of pregnancies with a recorded outcome

Regression estimates are on the 0–1 probability scale, and they should be multiplied by 100 to translate into rates on the percentage scale presented in the descriptive appendix tables

** p < 0.01, * p < 0.05

^¥^ Statistically significant after Bonferroni correction

**Table 2:** Risk of stillbirth (fetal death at/after 22 gestational weeks) across years with 2019 as the reference year

	January	February	March	April	May	June	July	August	September	October	November	December
Year	Regression estimates (Standard errors)											
2016	-0.001	-0.000	0.000	0.000	-0.002**	0.001	-0.000	-0.001*	-0.000	-0.000	0.000	-0.001
	(0.001)	(0.001)	(0.001)	(0.001)	(0.001)	(0.001)	(0.001)	(0.001)	(0.001)	(0.001)	(0.001)	(0.001)
2017	-0.000	-0.000	0.000	-0.000	-0.001	-0.000	-0.000	-0.001	0.000	-0.000	-0.001	-0.001
	(0.001)	(0.001)	(0.001)	(0.001)	(0.001)	(0.001)	(0.001)	(0.001)	(0.001)	(0.001)	(0.001)	(0.001)
2018	0.000	0.000	0.001	-0.000	-0.001*	-0.000	0.000	0.000	0.000	0.000	0.000	-0.001
	(0.001)	(0.001)	(0.001)	(0.001)	(0.001)	(0.001)	(0.001)	(0.001)	(0.001)	(0.001)	(0.001)	(0.001)
2019	Ref.	Ref.	Ref.	Ref.	Ref.	Ref.	Ref.	Ref.	Ref.	Ref.	Ref.	Ref.
2020	0.001	0.000	0.001*	0.001	0.001	0.001*	0.001**	0.001*	0.000	-0.000	-0.000	-0.000
	(0.001)	(0.001)	(0.001)	(0.001)	(0.001)	(0.001)	(0.001)	(0.001)	(0.001)	(0.001)	(0.001)	(0.001)
Observations	262,061	238,129	261,721	252,550	260,753	251,168	263,043	270,018	282,787	273,126	262,507	262,147

Estimates are rounded to the third decimal and are estimated relative to the total number of pregnancies with a recorded outcome

Regression estimates are on the 0–1 probability scale, and they should be multiplied by 100 to translate into rates on the percentage scale presented in the descriptive appendix tables

** p < 0.01, * p < 0.05

^¥^ Statistically significant after Bonferroni correction

### Study variables

Three binary (0/1) indicators for fetal deaths were examined: miscarriages (fetal deaths before 22 gestational weeks), stillbirths (fetal deaths at/after 22 gestational weeks), and fetal deaths without data on gestational age. Birth weight and gestational age are only provided as categorical measures in the publicly available birth certificate dataset, with gestational age being based on the date of the last menstrual period. Therefore, we examined birth weight as an interval variable (with 8 categories < 1000, 1000–1499, 1500–1999, 2000–2499, 2500–2999, 3000–3499, 3500–3999, ≥ 4000) measured in 500-grams intervals, and a binary (0/1) indicator of gestational age at or below 37 weeks. In the birth certificate dataset, the gestational age of 37 weeks is combined with 28–36 weeks in one category. We also evaluated the number of prenatal visits as a count variable, and caesarean delivery as a binary (0/1) indicator. In the birth certificate dataset, the type of delivery is a measure with three categories of normal, caesarean, and with instruments. Delivery with instruments accounted for less than 1.5% of births during the study period, so we decided to focus on caesarean delivery as one of the study outcomes.

We included several control variables in our analysis. Socio-demographic factors were maternal age, educational level, and marital status. We also included type of health insurance with categories for contributory (including exceptional), subsidised, and uninsured. In Colombia, individuals and families are enrolled in health insurance plans according to their working status and the household poverty level [24, 25]. We also controlled for place of residence (urban/rural), municipality of birth, and the number of pregnancies the mother has had before last pregnancy. Control variables were derived from the birth or death certificate records and included using the same categories presented in Appendix Table 3.

**Table 3:** Differences across years in birth weight measured in 500-gram intervals for live births (2019 is the reference year)

	January	February	March	April	May	June	July	August	September	October	November	December
Year	Regression estimates (Standard errors)											
2016	0.013*	0.019**	0.041**^¥^	0.007	0.030**^¥^	0.013*	0.022**^¥^	0.050**^¥^	0.045**^¥^	0.049**^¥^	0.040**^¥^	0.054**^¥^
	(0.006)	(0.007)	(0.006)	(0.007)	(0.006)	(0.007)	(0.006)	(0.006)	(0.006)	(0.006)	(0.006)	(0.006)
2017	0.005	0.031**^¥^	0.031**^¥^	0.019**	0.023**^¥^	0.034**^¥^	0.028**^¥^	0.045**^¥^	0.027**^¥^	0.040**^¥^	0.034**^¥^	0.054**^¥^
	(0.006)	(0.007)	(0.006)	(0.007)	(0.006)	(0.007)	(0.006)	(0.006)	(0.006)	(0.006)	(0.006)	(0.006)
2018	0.008	0.006	0.021**^¥^	0.017**	0.026**^¥^	0.017**	0.022**^¥^	0.027**^¥^	0.015*	0.020**	0.021**^¥^	0.033**^¥^
	(0.006)	(0.007)	(0.006)	(0.007)	(0.006)	(0.007)	(0.006)	(0.006)	(0.006)	(0.006)	(0.006)	(0.006)
2019	Ref.	Ref.	Ref.	Ref.	Ref.	Ref.	Ref.	Ref.	Ref.	Ref.	Ref.	Ref.
2020	-0.032**	-0.017*	0.008	0.031**^¥^	0.042**^¥^	0.039**^¥^	0.025**^¥^	0.024**^¥^	0.025**^¥^	0.025**^¥^	0.029**^¥^	0.034**^¥^
	(0.006)	(0.007)	(0.007)	(0.007)	(0.007)	(0.007)	(0.006)	(0.006)	(0.006)	(0.006)	(0.006)	(0.006)
Observations	247,902	224,528	247,423	239,244	246,938	238,261	250,048	256,767	269,938	260,344	250,148	249,693

Regression estimates are on the 0–1 probability scale, and they can be multiplied by 500 to approximate the differences in grams

Estimates are rounded to the third decimal

** p < 0.01, * p < 0.05

^¥^ Statistically significant after Bonferroni correction

### Statistical analysis

Linear regression models were used to compare outcomes month by month between 2019, as the reference year, the pandemic year 2020, and 2016, 2017 and 2018. This month-by-month comparison allowed for examining how the pandemic effects might have changed over time in 2020 (partly due to differences in duration of exposure to the pandemic in 2020 or due to changes in the mechanisms discussed above) while also accounting for seasonal differences in outcomes. Including data before 2019 allowed for examining whether changes in 2020 are new or reflecting prior trends. Separate regression models were estimated for each outcome and month. In addition to the covariates described above, the models included the year variable (with 2019 as reference). We used Bonferroni correction to evaluate the statistical significance of estimates given the multiple comparisons. The corrected critical p value (0.00104) was derived dividing 0.05 by 48, as we analysed 4 main outcomes across 12 months each. All analyses were conducted in Stata V.17.

The study utilizes publicly available and de-identified dataset, so ethics committee review and approval were not required.

## Results

### Sample description

The demographic and socioeconomic characteristics of all pregnancies included in the analysis are presented in Appendix Table 3. In 2020, 4.1% of pregnancies ended up in fetal deaths (3.0% miscarriages, 0.8% stillbirths, and 0.3% fetal deaths without data on gestational age) (Appendix Table 4). Among live births, in 2020 the majority weighted between 3000 and 3499 g (43.4%), followed by 2500–2999 g (27.6%) and 3500–3999 g (18.0%); 8.1% had a birth weight below 2500 g. About 20.2% had a gestational age at or below 37 weeks. The mean number of prenatal visits was 5.8 (SD = 2.7), and 44.5% had a caesarean section (Appendix Table 4).

**Table 4:** Differences across years in number of prenatal visits obtained throughout pregnancy for live births (2019 is the reference year)

	January	February	March	April	May	June	July	August	September	October	November	December
Year	Regression estimates (Standard errors)											
2016	0.073**^¥^	0.036*	0.061**^¥^	0.018	0.086**^¥^	0.108**^¥^	0.104**^¥^	0.123**^¥^	0.140**^¥^	0.114**^¥^	0.124**^¥^	0.123**^¥^
	(0.014)	(0.015)	(0.015)	(0.015)	(0.014)	(0.015)	(0.015)	(0.014)	(0.014)	(0.014)	(0.015)	(0.014)
2017	0.071**^¥^	0.014	0.031*	-0.011	0.016	-0.007	-0.013	-0.007	-0.003	0.004	0.024	0.055**^¥^
	(0.014)	(0.015)	(0.015)	(0.015)	(0.014)	(0.015)	(0.014)	(0.014)	(0.014)	(0.014)	(0.014)	(0.014)
2018	-0.007	-0.011	-0.004	-0.043**	0.044**	0.043**	0.001	-0.001	0.001	0.001	0.031*	0.043**
	(0.014)	(0.015)	(0.015)	(0.015)	(0.015)	(0.015)	(0.014)	(0.014)	(0.014)	(0.014)	(0.014)	(0.014)
2019	Ref.	Ref.	Ref.	Ref.	Ref.	Ref.	Ref.	Ref.	Ref.	Ref.	Ref.	Ref.
2020	-0.012	-0.085**^¥^	-0.095**^¥^	-0.315**^¥^	-0.461**^¥^	-0.510**^¥^	-0.583**^¥^	-0.590**^¥^	-0.629**^¥^	-0.606**^¥^	-0.541**^¥^	-0.355**^¥^
	(0.014)	(0.015)	(0.015)	(0.015)	(0.015)	(0.015)	(0.015)	(0.015)	(0.014)	(0.014)	(0.014)	(0.015)
Observations	247,902	224,528	247,423	239,244	246,938	238,261	250,048	256,767	269,938	260,344	250,148	249,693

Estimates are rounded to the third decimal

** p < 0.01, * p < 0.05

^¥^ Statistically significant after Bonferroni correction

### Fetal deaths

Table 1 reports the month-by-month differences across years in risk of miscarriages (fetal deaths before 22 gestational weeks), with 2019 as the reference year. The estimates are from the regression of the likelihood of a miscarriage using individual-level data combining birth and death certificates, so the probability is estimated relative to the total number of pregnancies with a recorded outcome. In 2020, there was a decline (at p < 0.05 or less) in miscarriage risk compared to 2019 beginning in April through September and November, with estimates remaining statistically significant after Bonferroni correction for May, July-September, and November. There was no difference between 2020 and 2019 in January-March, October, and December. For May-July, there were no clear pre-pandemic trends in miscarriage risk comparing 2019 to 2016–2018. For those months, miscarriage risk was lower in 2020 compared to 2019 by 2.7 to 9.1 per 1000 pregnancies. However, for March-April, and August estimates show that the decrease in the risk of miscarriage had already started in 2019.

In Table 2, we show the estimates from a similar model for the risk of stillbirth (fetal death at/after 22 gestational weeks). In 2020, there was a higher risk in March and June-August compared to 2019 by 1.2 stillbirths per 1000 pregnancies (at p < 0.05 or less); however, the estimates were no longer statistically significant after Bonferroni correction. For these months, there was no difference of pre-pandemic trends comparing 2019 to 2016–2018. There was no difference between 2020 and 2019 in the other months.

For fetal deaths without information about gestational age, declines were observed in January, March, July-August, and November in 2020 compared to 2019 (Appendix Table 5); only the estimate for March (showing a higher risk by 1.7 stillbirths per 1000 pregnancies) remained statistically significant after Bonferroni correction. For those months however, there is some evidence of previous trends in declining risk of fetal deaths comparing 2019 to 2016–2018.

### Birth weight and gestational age

Table 3 reports the month-by-month differences across years in birth weight measured in 500-gram intervals for live births. Estimates can be multiplied by 500 to approximate the differences in grams. Mean birth weight was higher in 2020 than 2019 for births in April through December by about 12 to 21 g (p < 0.01), with estimates remaining statistically significant after Bonferroni correction. For these months, mean birth weight was generally declining before 2020, with a higher mean birth weight in 2016–2018 than 2019, indicating an opposite trend after the pandemic in 2020. The pre-pandemic trend of declining birth weight across years appeared to continue in January and February of 2020 before the pandemic.

Appendix Table 6 shows the estimates for likelihood of gestational age at or below 37 weeks. In April-June, there was a lower risk of gestational age at/below 37 weeks in 2020 than 2019 (p < 0.01). In all months, there was a pre-pandemic trend of increasing likelihood of 37 weeks or lower gestational age (comparing 2016–2018 to 2019), so the difference in April-June between 2020 and 2019 represents an opposite trend to the pre-pandemic trend. In contrast to these four months, this likelihood is higher in October 2020 than 2019, consistent with the pre-pandemic trend. After the Bonferroni correction, differences between 2020 and 2019 remained statistically significant for April, June and October. For April and June, the risk of gestational age at or below 37 weeks was lower in 2020 compared to 2019 by 13.7 and 9.5 per 1000 pregnancies, respectively.

### Prenatal visits and C-Sections

Table 4 reports the month-by-month differences across years in number of prenatal visits obtained throughout pregnancy for live births. In 2020, there was a noticeable decline in prenatal visits beginning with births in April and throughout the year compared to 2019, with the largest difference in June-October by 0.5–0.6 visits per birth on average or up to 10% of the mean number of prenatal visits in 2019. There was also a much smaller decline in February-March. For some months, there is some evidence of pre-pandemic trends in declining prenatal visits over years, however the magnitude of these trends is much smaller than the difference between 2019 and 2020. Collectively, these results suggest reduced prenatal care utilization following the pandemic start. All differences observed remained significant after the Bonferroni correction.

Appendix Table 7 shows the estimates for likelihood of C-section deliveries among live births. Following the pandemic start, the likelihood of C-sections was higher in June-July and September-December than 2019. However, in most of these months, this difference appears to reflect a pre-pandemic trend. Differences between 2019 and 2020 remained significant after the Bonferroni correction for July, September and October. There was also a higher likelihood of C-sections in February in 2020 than 2019, which would not be related to the pandemic since that month precedes the pandemic onset in Colombia. Taken together, these results suggest little evidence for an effect of the pandemic on C-sections.

## Discussion

This study examined changes in perinatal health outcomes and prenatal care in Colombia following the COVID-19 pandemic in 2020. Using national population-based registers, we find some evidence for a decline in miscarriage risk especially in 2–4 months (May-July) after the pandemic start on March 2020, a period when the lockdowns restrictions were introduced. In contrast, there is an apparent lagging increase in stillbirth risk over this period, although estimates are not statistically significant after correction for multiple comparisons. At the same time, there is an increase in birth weight throughout 2020 following the pandemic start over much of the post-pandemic period that does not appear to be driven by pre-pandemic trends. There is also evidence of decline in prenatal visits especially in June-October, but no evidence of a change in C-section delivery following the pandemic. Taken together, the findings suggest mixed effects of the pandemic on perinatal outcomes and underlying pathways, highlighting the importance of studying multiple outcomes to understand impacts on maternal and child health and more broadly on population health.

In Colombia, death certificates are completed by health professionals providing care in the event of a fetal death recorded anytime during pregnancy. In this study, the observed decline in the risk of miscarriage in May-July might reflect underreporting due to reduced access to health care services. It might, however, be related to other factors including changes in maternal exposure to job hazards and stress. At the same time, the possible increase in stillbirth risk might be related to maternal COVID-19 infections[26] and the decline in prenatal care utilization since stillbirth risk is more influenced by prenatal care, while the risk of miscarriage has stronger associations with pre-conception factors [27–29].

There are various factors to consider when trying to understand the increase in birth weight average observed after the pandemic start in 2020. The increase could not be explained by the increase in stillbirth risk as the increase in birth weight was observed in all 9 months (April-December) in 2020 after the pandemic start, while the stillbirth risk increase was observed in 4 months (March, and June-August), suggesting that other factors may contribute to the birth weight difference. Furthermore, considering the number of stillbirths who would have been born alive with lower birth weight (about 263 for July, for example), the potential change in the mean birth weight would be very small to account for the observed change in birth weight mean. Also, the differences in likelihood of gestational age at/below 37 weeks across months suggest that birth weight differences are perhaps mostly due to changes in the fetal growth rate rather than differences in gestational age. However, given the limitation in measuring gestational age in the publicly available data, we cannot reliably separate changes due to gestational age versus fetal growth rate. Moreover, the simultaneous decline in prenatal visits and increase in birth weight should not be interpreted that the number of prenatal visits has no effect on birth weight; multiple studies from South American settings suggest benefits to birth weight with more prenatal care use [30–33]. Instead, the observed increase in birth weight might reflect other changes that might have offset the negative effects of decline in prenatal care, such as reduced maternal exposure to air pollution, occupational health hazards, and job stress [6, 8]. These factors could have also counterbalanced the negative effects of the lower number of prenatal visits if some alternative prenatal care was provided, e.g., through virtual appointments, home visits, etc. In addition, the observed increase in stillbirths could have resulted in better outcomes on average among live births.

The study has multiple strengths including national registers that cover almost all births in Colombia, the use of multiple perinatal outcomes, a month-by-month comparison to account for differential exposures to the pandemic in 2020 by length and time of pregnancy, and including multiple years to examine pre-pandemic trends. There are also caveats to consider when interpreting the results. The data on fetal death certificates likely misses a proportion of fetal deaths especially early during the pregnancy. As such, the observed decline in early fetal deaths (before 22 gestational weeks) could partly reflect more underreporting few months following the pandemic start as noted above. There was also no detailed data on gestational age to further understand changes in preterm birth or late term delivery, which may also relate to the higher observed birth weight. It is worth noting that a causal relationship is very difficult to establish in this case given the various and complex mechanisms behind the perinatal outcomes analysed and the multiple unknown effects that the pandemic could have had on different aspects related to the pregnancies, mothers and families. Finally, we had no readily available data to examine potential underlying mechanisms, including specific measures of the lockdowns or restrictions on access to health care. In Colombia, most of the policies were national, although there were some local variations. Understanding how local differences in restrictions and economic impacts of the pandemic affected perinatal outcomes is an important future research endeavour.

To conclude, this study provides evidence on some of the possible early effects of the pandemic on perinatal health in Colombia. Specifically, our findings suggest that following the pandemic onset in Colombia, an increase in mean birth weight, a decline in miscarriage risk, and fewer prenatal visits were observed. There was also a lower risk of gestational age at/below 37 weeks for two months and no significant changes in C-sections. Examining these effects in subsequent years as data become available is critical to understanding the pandemic’s short and long-term effects on a key domain of population health. In addition, analysing how these effects differ across socioeconomic and ethnic subgroups would shed light on the combined potential effects of the pandemic and the social determinants of perinatal health. The study findings also highlight the need for continuing and where possible intensifying perinatal health surveillance to capture the multiple outcomes that could be affected including those that may become underreported during the pandemic such as early fetal deaths.

## Electronic supplementary material

Below is the link to the electronic supplementary material.

Supplementary Material 1

## Data Availability

All birth certificate data in Colombia are stored in an open-access database available through the DANE website (https://www.dane.gov.co).

## List of Abbreviations

CI: Confidence interval
C-section: Caesarean delivery
DANE: Colombian National Department of Statistics (Acronym in Spanish)
SD: Standard deviation
