# Supplementary material for: Changes in birth outcomes and utilization of prenatal care during the COVID-19 pandemic in 2020: a secondary analysis of vital statistics in Colombia
Source: BMC Pediatr. 2023 May 12;23:234. doi: 10.1186/s12887-023-04027-9 (PMC10175897; doi:10.1186/s12887-023-04027-9)
Supplement: Supplementary file 1 — Supplementary Material 1 [file 12887_2023_4027_MOESM1_ESM.docx]

Appendix Table 1. Description of missing values per variables and years of full sample of pregnancies

|  | 2016 | 2017 | 2018 | 2019 | 2020 | Total |
| --- | --- | --- | --- | --- | --- | --- |
|  | N=674,107 | N=680,582 | N=671,392 | N=663,244 | N=646,376 | N=3,335,701 |
|  |  |  |  |  |  |  |
| Maternal age; n (%) | 122 (0.02) | 339 (0.05) | 965 (0.14) | 1,973 (0.3) | 2,382 (0.37) | 5,781 (0.17) |
| Education level; n (%) | 28,208 (4.18) | 26,107 (3.84) | 30,474 (4.54) | 30,071 (4.53) | 27,258 (4.22) | 142,118 (4.26) |
| Insurance scheme; n (%) | 184 (0.03) | 367 (0.05) | 324 (0.05) | 299 (0.05) | 207 (0.03) | 1,381 (0.04) |
| Location of residence; n (%) | 561 (0.08) | 1,582 (0.23) | 4,551 (0.68) | 12,232 (1.84) | 10,254 (1.59) | 29,180 (0.87) |
| Marital state; n (%) | 17,921 (2.66) | 18,320 (2.69) | 21,946 (3.27) | 23,553 (3.55) | 20,826 (3.22) | 102,566 (3.07) |
| Number of children (including this one); n (%) | 158 (0.02) | 353 (0.05) | 295 (0.04) | 309 (0.05) | 208 (0.03) | 1,323 (0.04) |
| Municipality; n (%) | 0 (0.00) | 0 (0.00) | 0 (0.00) | 12,231 (1.84) | 10,250 (1.59) | 22,481 (0.67) |
|  |  |  |  |  |  |  |
| Missing in all variables; n (%) | 33,393 (4.95) | 33,926 (4.98) | 40,192 (5.99) | 45,993 (6.93) | 42,187 (6.53) | 195,691 (5.87) |
|  |  |  |  |  |  |  |

Appendix Table 2. Description of missing values per variables and years of full sample of live births

|  | 2016 | 2017 | 2018 | 2019 | 2020 | Total |
| --- | --- | --- | --- | --- | --- | --- |
|  | N=636,076 | N=645,269 | N=637,717 | N=631,546 | N=618,330 | N=3,168,938 |
|  |  |  |  |  |  |  |
| Maternal age; n (%) | 73 (0.01) | 69 (0.01) | 670 (0.11) | 1,684 (0.27) | 2,177 (0.35) | 4,673 (0.15) |
| Education level; n (%) | 24,211 (3.81) | 22,751 (3.53) | 27,100 (4.25) | 26,512 (4.20) | 25,074 (4.06) | 125,648 (3.96) |
| Insurance scheme; n (%) | 138 (0.02) | 74 (0.01) | 1 (0.00) | 0 (0.00) | 0 (0.00) | 213 (0.01) |
| Location of residence; n (%) | 452 (0.07) | 1,272 (0.20) | 4,006 (0.63) | 11,353 (1.80) | 9,606 (1.55) | 26,689 (0.84) |
| Marital state; n (%) | 14,841 (2.33) | 15,818 (2.45) | 19,252 (3.02) | 20,556 (3.25) | 19,047 (3.08) | 89,514 (2.82) |
| Number of children (including this one); n (%) | 74 (0.01) | 74.00 (0.01) | 11 (0.00) | 18.00 (0.00) | 6 (0.00) | 183 (0.01) |
| Municipality; n (%) | 0 (0.00) | 0 (0.00) | 0 (0.00) | 11,353 (1.80) | 9,605 (1.55) | 20,958 (0.66) |
| Gestational age | 2,023 (0.32) | 1,903 (0.29) | 4,994 (0.78) | 3,858 (0.61) | 8,390 (1.36) | 21,168 (0.67) |
| Birth weight | 1,751 (0.28) | 1,903 (0.29) | 4,974 (0.78) | 4,068 (0.64) | 8,304 (1.34) | 21,000 (0.66) |
| Prenatal Visits | 1,194 (0.19) | 1,186 (0.18) | 3,842 (0.60) | 3,125 (0.49) | 7,489 (1.21) | 16,836 (0.53) |
| C-sections | 16 (0.00) | 6 (0.00) | 0 (0.00) | 0 (0.00) | 0 (0.00) | 22 (0.00) |
|  |  |  |  |  |  |  |
| Missing in all variables; n (%) | 30,859 (4.85) | 32,161 (4.98) | 38,518 (6.04) | 43,594 (6.90) | 42,572 (6.88) | 187,704 (5.92) |
|  |  |  |  |  |  |  |

Appendix Table 3. Baseline characteristics of the full sample of pregnancies (excluding individuals with missing values)

|  | 2016 | 2017 | 2018 | 2019 | 2020 | Total |
| --- | --- | --- | --- | --- | --- | --- |
|  | N=640,714 | N=646,656 | N=631,200 | N=617,251 | N=604,189 | N=3,140,010 |
| Maternal age in categories; n (%) |  |  |  |  |  |  |
| 14 or less | 5,549 (0.87) | 5,773 (0.89) | 5,319 (0.84) | 4,572 (0.74) | 4,134 (0.68) | 25,347 (0.81) |
| 15-19 | 127,628 (19.92) | 125,613 (19.43) | 118,948 (18.84) | 111,560 (18.07) | 105,016 (17.38) | 588,765 (18.75) |
| 20-24 | 185,736 (28.99) | 186,362 (28.82) | 180,564 (28.61) | 176,334 (28.57) | 174,450 (28.87) | 903,446 (28.77) |
| 25-29 | 149,064 (23.27) | 152,242 (23.54) | 151,986 (24.08) | 151,180 (24.49) | 150,633 (24.93) | 755,105 (24.05) |
| 30-34 | 103,394 (16.14) | 104,033 (16.09) | 102,173 (16.19) | 102,210 (16.56) | 100,110 (16.57) | 511,920 (16.3) |
| 35-39 | 54,473 (8.5) | 57,273 (8.86) | 56,542 (8.96) | 55,847 (9.05) | 54,278 (8.98) | 278,413 (8.87) |
| 40 or more | 14,870 (2.32) | 15,360 (2.38) | 15,668 (2.48) | 15,548 (2.52) | 15,568 (2.58) | 77,014 (2.45) |
| Education level; n (%) |  |  |  |  |  |  |
| University | 79,511 (12.41) | 79,535 (12.30) | 78,605 (12.45) | 78,586 (12.73) | 75,067 (12.42) | 391,304 (12.46) |
| Technical | 92,105 (14.38) | 97,803 (15.12) | 99,100 (15.70) | 97,637 (15.82) | 95,156 (15.75) | 481,801 (15.34) |
| Secondary | 376,491 (58.76) | 379,430 (58.68) | 367,390 (58.21) | 359,682 (58.27) | 355,217 (58.79) | 1,838,210 (58.54) |
| Primary or less | 92,607 (14.45) | 89,888 (13.90) | 86,105 (13.64) | 81,346 (13.18) | 78,749 (13.03) | 428,695 (13.65) |
| Insurance scheme; n (%) |  |  |  |  |  |  |
| Contri/Exc/Spec | 302,146 (47.16) | 288,157 (44.56) | 265,218 (42.02) | 246,361 (39.91) | 232,485 (38.48) | 1,334,367 (42.50) |
| Subsidized | 323,033 (50.42) | 341,704 (52.84) | 334,197 (52.95) | 319,284 (51.73) | 311,911 (51.62) | 1,630,129 (51.91) |
| Uninsured | 15,535 (2.42) | 16,795 (2.60) | 31,785 (5.04) | 51,606 (8.36) | 59,793 (9.90) | 175,514 (5.59) |
| Location of residence; n (%) |  |  |  |  |  |  |
| Urban | 511985 (79.91) | 510,864 (79.00) | 497,436 (78.81) | 487,904 (79.04) | 471,146 (77.98) | 2479335 (78.96) |
| Small villages | 46525 (7.26) | 49,478 (7.65) | 46,430 (7.36) | 43,982 (7.13) | 43,336 (7.17) | 229751 (7.32) |
| Rural | 82204 (12.83) | 86,314 (13.35) | 87,334 (13.84) | 85,365 (13.83) | 89,707 (14.85) | 430924 (13.72) |
| Marital state; n (%) |  |  |  |  |  |  |
| Married or in consensus | 542,548 (84.68) | 550,562 (85.14) | 538,066 (85.24) | 528,372 (85.60) | 521,062 (86.24) | 2,680,610 (85.37) |
| Divorced or widowed | 2,678 (0.42) | 2,619 (0.41) | 2,559 (0.41) | 2,251 (0.36) | 2,194 (0.36) | 12,301 (0.39) |
| Single | 95,488 (14.90) | 93,475 (14.46) | 90,575 (14.35) | 86,628 (14.03) | 80,933 (13.40) | 447,099 (14.24) |
| Number of children (including this one); n (%) |  |  |  |  |  |  |
| One | 317,385 (49.54) | 316,424 (48.93) | 302,366 (47.90) | 288,990 (46.82) | 278,613 (46.11) | 1,503,778 (47.89) |
| Two | 200,532 (31.30) | 205,432 (31.77) | 203,078 (32.17) | 202,124 (32.75) | 199,500 (33.02) | 1,010,666 (32.19) |
| Three | 75,997 (11.86) | 77,742 (12.02) | 78,801 (12.48) | 79,522 (12.88) | 79,772 (13.20) | 391,834 (12.48) |
| Four | 25,429 (3.97) | 26,031 (4.03) | 26,096 (4.13) | 26,171 (4.24) | 26,377 (4.37) | 130,104 (4.14) |
| Five or more | 21,371 (3.34) | 21,027 (3.25) | 20,859 (3.30) | 20,444 (3.31) | 19,927 (3.30) | 103,628 (3.30) |

Appendix Table 4. Outcomes by year

|  | 2016 | 2017 | 2018 | 2019 | 2020 | Total |
| --- | --- | --- | --- | --- | --- | --- |
|  |  |  |  |  |  |  |
| Deaths |  |  |  |  |  |  |
| Fetal deaths; n (%) | 33,394 (5.21) | 31,433 (4.86) | 29,508 (4.67) | 27,111 (4.39) | 25,030 (4.14) | 146,476 (4.66) |
| Miscarriages; n (%) | 23,021 (3.59) | 22,427 (3.47) | 21,711 (3.44) | 19,947 (3.23) | 17,960 (2.97) | 105,066 (3.35) |
| Stillbirths; n (%) | 4,572 (0.71) | 4,678 (0.72) | 4,821 (0.76) | 4,820 (0.78) | 5,121 (0.85) | 24,012 (0.76) |
| Unknown; n (%) | 5,801 (0.91) | 4,328 (0.67) | 2,976 (0.47) | 2,344 (0.38) | 1,949 (0.32) | 17,398 (0.55) |
|  |  |  |  |  |  |  |
| Neonatal outcomes |  |  |  |  |  |  |
| Preterm births; n (%) | 114,790 (18.95) | 117,261 (19.11) | 116,803 (19.47) | 119,415 (20.28) | 116,641 (20.21) | 584,910 (19.59) |
| Low birthweight; n (%) | 48,693 (8.04) | 49,293 (8.03) | 48,773 (8.13) | 49,386 (8.39) | 46,681 (8.1) | 242,826 (8.14) |
| Birthweight categories; n (%) |  |  |  |  |  |  |
| Lower than 1000 gr | 2,205 (0.36) | 2,116 (0.34) | 2,140 (0.36) | 2,040 (0.35) | 1,928 (0.33) | 10,429 (0.35) |
| 1000 to 1499 | 3,851 (0.64) | 3,799 (0.62) | 3,735 (0.62) | 3,685 (0.63) | 3,493 (0.61) | 18,563 (0.62) |
| 1500 to 1999 | 8,327 (1.37) | 8,497 (1.38) | 8,241 (1.37) | 8,337 (1.42) | 7,846 (1.36) | 41,248 (1.38) |
| 2000 to 2499 | 34,310 (5.66) | 34,881 (5.68) | 34,657 (5.78) | 35,324 (6) | 33,414 (5.8) | 172,586 (5.78) |
| 2500 to 2999 | 166,181 (27.43) | 167,333 (27.27) | 165,131 (27.53) | 165,559 (28.14) | 159,215 (27.62) | 823,419 (27.59) |
| 3000 to 3499 | 263,706 (43.52) | 267,131 (43.53) | 261,061 (43.52) | 255,281 (43.38) | 250,281 (43.42) | 1,297,460 (43.48) |
| 3500 to 3999 | 110,234 (18.19) | 112,093 (18.27) | 108,054 (18.01) | 102,968 (17.5) | 103,997 (18.04) | 537,346 (18.01) |
| 4000 or more | 17,108 (2.82) | 17,848 (2.91) | 16,791 (2.8) | 15,221 (2.59) | 16,288 (2.83) | 83,256 (2.79) |
|  |  |  |  |  |  |  |
| Access outcomes |  |  |  |  |  |  |
| C-section; n (%) | 272,444 (44.86) | 273,616 (44.47) | 263,032 (43.72) | 260,602 (44.16) | 257,573 (44.47) | 1,327,267 (44.34) |
| Prenatal visits; Mean (SD) | 6.51 (2.55) | 6.42 (2.55) | 6.33 (2.63) | 6.24 (2.69) | 5.79 (2.69) | 6.26 (2.63) |
|  |  |  |  |  |  |  |
| Population |  |  |  |  |  |  |
| Total of pregnancies | 640,714 | 646,656 | 631,200 | 617,251 | 604,189 | 3,140,010 |
| Total of live births | 607,320 | 615,223 | 601,692 | 590,140 | 579,159 | 2,993,534 |

Appendix Table 5. Risk of fetal death across years with 2019 as the reference year.

|  | January | February | March | April | May | June | July | August | September | October | November | December |
| --- | --- | --- | --- | --- | --- | --- | --- | --- | --- | --- | --- | --- |
| Year | Regression estimates (Standard errors) | | | | | | | | | | | |
|  |  |  |  |  |  |  |  |  |  |  |  |  |
| 2016 | 0.005**^¥^ | 0.008**^¥^ | 0.004**^¥^ | 0.007**^¥^ | 0.005**^¥^ | 0.006**^¥^ | 0.004**^¥^ | 0.006**^¥^ | 0.006**^¥^ | 0.006**^¥^ | 0.005**^¥^ | 0.005**^¥^ |
|  | (0.000) | (0.001) | (0.000) | (0.000) | (0.000) | (0.000) | (0.000) | (0.000) | (0.000) | (0.000) | (0.000) | (0.000) |
| 2017 | 0.003**^¥^ | 0.006**^¥^ | 0.003**^¥^ | 0.005**^¥^ | 0.004**^¥^ | 0.005**^¥^ | 0.002**^¥^ | 0.004**^¥^ | 0.001**^¥^ | 0.002**^¥^ | 0.001** | 0.001**^¥^ |
|  | (0.000) | (0.001) | (0.000) | (0.000) | (0.000) | (0.000) | (0.000) | (0.000) | (0.000) | (0.000) | (0.000) | (0.000) |
| 2018 | 0.000 | 0.002**^¥^ | -0.001 | 0.001** | 0.002**^¥^ | 0.002**^¥^ | 0.001 | 0.002**^¥^ | 0.001** | 0.001** | -0.000 | 0.001 |
|  | (0.000) | (0.001) | (0.000) | (0.000) | (0.000) | (0.000) | (0.000) | (0.000) | (0.000) | (0.000) | (0.000) | (0.000) |
| 2019 | Ref. | Ref. | Ref. | Ref. | Ref. | Ref. | Ref. | Ref. | Ref. | Ref. | Ref. | Ref. |
|  |  |  |  |  |  |  |  |  |  |  |  |  |
| 2020 | -0.001* | 0.000 | -0.002**^¥^ | -0.000 | -0.000 | -0.000 | -0.001** | -0.001* | -0.001 | -0.000 | -0.001** | 0.000 |
|  | (0.000) | (0.001) | (0.001) | (0.001) | (0.000) | (0.000) | (0.000) | (0.000) | (0.000) | (0.000) | (0.000) | (0.000) |
|  |  |  |  |  |  |  |  |  |  |  |  |  |
| Observations | 262,061 | 238,129 | 261,721 | 252,550 | 260,753 | 251,168 | 263,043 | 270,018 | 282,787 | 273,126 | 262,507 | 262,147 |
|  |  |  |  |  |  |  |  |  |  |  |  |  |

Estimates are rounded to the third decimal and are estimated relative to the total number of pregnancies with a recorded outcome.

Regression estimates are on the 0-1 probability scale, and they should be multiplied by 100 to translate into rates on the percentage scale presented in the descriptive appendix tables.

** p<0.01, * p<0.05

^¥^ Statistically significant after Bonferroni correction.

Appendix Table 6. Risk of preterm birth (live birth with ≤37 weeks of pregnancy) across years with 2019 as the reference year.

|  | January | February | March | April | May | June | July | August | September | October | November | December |
| --- | --- | --- | --- | --- | --- | --- | --- | --- | --- | --- | --- | --- |
| Year | Regression estimates (Standard errors) | | | | | | | | | | | |
|  |  |  |  |  |  |  |  |  |  |  |  |  |
| 2016 | -0.014** | -0.005* | -0.016**^¥^ | -0.009**^¥^ | -0.012**^¥^ | -0.016**^¥^ | -0.012**^¥^ | -0.019**^¥^ | -0.021**^¥^ | -0.016**^¥^ | -0.008**^¥^ | -0.019**^¥^ |
|  | (0.002) | (0.003) | (0.003) | (0.003) | (0.003) | (0.003) | (0.003) | (0.003) | (0.002) | (0.003) | (0.003) | (0.003) |
| 2017 | -0.005* | -0.007** | -0.014**^¥^ | -0.006* | -0.011**^¥^ | -0.017**^¥^ | -0.007** | -0.015**^¥^ | -0.013**^¥^ | -0.013**^¥^ | -0.013**^¥^ | -0.021**^¥^ |
|  | (0.003) | (0.003) | (0.003) | (0.003) | (0.002) | (0.003) | (0.003) | (0.003) | (0.002) | (0.002) | (0.003) | (0.003) |
| 2018 | -0.011**^¥^ | -0.004 | -0.008**^¥^ | -0.002 | -0.009**^¥^ | -0.010**^¥^ | -0.008**^¥^ | -0.011**^¥^ | -0.008** | -0.008** | -0.003 | -0.013**^¥^ |
|  | (0.003) | (0.003) | (0.003) | (0.003) | (0.003) | (0.003) | (0.003) | (0.003) | (0.002) | (0.002) | (0.003) | (0.003) |
| 2019 | Ref. | Ref. | Ref. | Ref. | Ref. | Ref. | Ref. | Ref. | Ref. | Ref. | Ref. | Ref. |
|  |  |  |  |  |  |  |  |  |  |  |  |  |
| 2020 | 0.003 | 0.012**^¥^ | -0.001 | -0.014**^¥^ | -0.008** | -0.009**^¥^ | -0.001 | 0.001 | 0.002 | 0.013**^¥^ | 0.002 | 0.002 |
|  | (0.003) | (0.003) | (0.003) | (0.003) | (0.003) | (0.003) | (0.003) | (0.003) | (0.002) | (0.002) | (0.003) | (0.003) |
|  |  |  |  |  |  |  |  |  |  |  |  |  |
| Observations | 247,902 | 224,528 | 247,423 | 239,244 | 246,938 | 238,261 | 250,048 | 256,767 | 269,938 | 260,344 | 250,148 | 249,693 |
|  |  |  |  |  |  |  |  |  |  |  |  |  |

Estimates are rounded to the third decimal and are estimated relative to the total number of pregnancies with a recorded outcome.

Regression estimates are on the 0-1 probability scale, and they should be multiplied by 100 to translate into rates on the percentage scale presented in the descriptive appendix tables.

** p<0.01, * p<0.05

^¥^ Statistically significant after Bonferroni correction.

Appendix Table 7. Risk of C-section across years with 2019 as the reference year.

|  | January | February | March | April | May | June | July | August | September | October | November | December |
| --- | --- | --- | --- | --- | --- | --- | --- | --- | --- | --- | --- | --- |
| Year | Regression estimates (Standard errors) | | | | | | | | | | | |
|  |  |  |  |  |  |  |  |  |  |  |  |  |
| 2016 | 0.017**^¥^ | 0.021**^¥^ | 0.007* | 0.008** | 0.006* | 0.015** | 0.003 | 0.006* | 0.012**^¥^ | 0.012**^¥^ | 0.013**^¥^ | 0.009** |
|  | (0.003) | (0.003) | (0.003) | (0.003) | (0.003) | (0.003) | (0.003) | (0.003) | (0.003) | (0.003) | (0.003) | (0.003) |
| 2017 | 0.017**^¥^ | 0.009** | 0.015**^¥^ | 0.009** | 0.010**^¥^ | 0.008** | 0.002 | 0.006* | 0.010**^¥^ | -0.010**^¥^ | -0.006* | -0.019**^¥^ |
|  | (0.003) | (0.003) | (0.003) | (0.003) | (0.003) | (0.003) | (0.003) | (0.003) | (0.003) | (0.003) | (0.003) | (0.003) |
| 2018 | -0.001 | 0.003 | -0.007* | -0.004 | -0.009** | -0.005 | -0.011**^¥^ | -0.009** | 0.001 | -0.000 | -0.002 | -0.010**^¥^ |
|  | (0.003) | (0.003) | (0.003) | (0.003) | (0.003) | (0.003) | (0.003) | (0.003) | (0.003) | (0.003) | (0.003) | (0.003) |
| 2019 | Ref. | Ref. | Ref. | Ref. | Ref. | Ref. | Ref. | Ref. | Ref. | Ref. | Ref. | Ref. |
|  |  |  |  |  |  |  |  |  |  |  |  |  |
| 2020 | 0.005 | 0.007* | 0.001 | 0.003 | -0.002 | 0.006* | 0.010**^¥^ | 0.005 | 0.012**^¥^ | 0.015**^¥^ | 0.009** | 0.009** |
|  | (0.003) | (0.003) | (0.003) | (0.003) | (0.003) | (0.003) | (0.003) | (0.003) | (0.003) | (0.003) | (0.003) | (0.003) |
|  |  |  |  |  |  |  |  |  |  |  |  |  |
| Observations | 247,902 | 224,528 | 247,423 | 239,244 | 246,938 | 238,261 | 250,048 | 256,767 | 269,938 | 260,344 | 250,148 | 249,693 |
|  |  |  |  |  |  |  |  |  |  |  |  |  |

Estimates are rounded to the third decimal and are estimated relative to the total number of pregnancies with a recorded outcome.

Regression estimates are on the 0-1 probability scale, and they should be multiplied by 100 to translate into rates on the percentage scale presented in the descriptive appendix tables.

** p<0.01, * p<0.05

^¥^ Statistically significant after Bonferroni correction.
